# Fracture of the Cartilaginous Portion of the Nasal Septum, Followed by Acute Traumatic Perichondritis

**Published:** 1891-10

**Authors:** Charles Dunbar Roy

**Affiliations:** Assistant to the Chair of Eye, Ear and Throat, Southern Medical College, Atlanta, Ga.


Vol. VIII. OCTOBER, 1891. No. 8.

TO CONTRIBUTORS.

Articles for publication must reach this office not later than 15th inst.; and are expected to be published exclusively in this Journal. Extra copies of Journal furnished contributors when requested. Reprints by special arrangement. Publication of an article does not necessarily imply endorsement of the views therein expressed.

Atlanta Medical and Surgical Journal, Post-office box 431.

### Original Communications.

### FRACTURE OF THE CARTILAGINOUS PORTION OF THE NASAL SEPTUM FOLLOWED BY ACUTE TRAUMATIC PERICHONDRITIS.

By CHARLES DUNBAR ROY, A. B., M. D.,

Assistant to the Chair of Eye, Ear and Throat, Southern Medical College, Atlanta, Ga.

The history of the following case which came under the care of Dr. A. G. Hobbs and myself was one to me of much interest, and through the kind permission of Dr. H. I am enabled to give to the readers of this journal a report of the same. Many injuries of the nose and its accessory cavities have come under my observation, but none of them ever presented the especial features of this one.

M. J., student, age 19, presented himself at the office for consultation Monday, May 18th. From him the following history was elicited. On the Friday previous, while engaged in a game of base ball, the ball accidentally slipped through his hands and struck him on the tip end of his nose. The inconvenience occasioned at the time was slight, there being but little pain and scarcely any bleeding. He retired that night feeling comfortable and enjoyed a good night's rest. On the following morning he found his nose much swollen and considerable interference with nasal respiration. On Saturday and Sunday the conditions remained the same. Hot water was used externally, in conjunction with the topical application of borax and vaseline. On Monday morning he presented himself at the office.

*Examination.*—By inspection the cartilaginous portion of the nose externally showed considerable swelling. By tilting up the anterior extremity and with the aid of reflected light, the nasal cavities could be brought into view, showing complete occlusion of the canals. The occlusion was formed by protrusions from each side of the cartilaginous portion of the septum. The patient could scarcely obtain a passage for air by the most strenuous exertion. The septal cartilage gave the appearanc of having been pulpified by the blow.

By palpation with the tips of my little fingers introduced into the canals, the condition of the septum was still further ascertained. The cartilage was easily movable to first one side and then the other, with an occasional exception. The anterior half would move without seeming disturbance of the posterior portion, though the swelling and œdema were so great as to make the manipulation somewhat difficult. By means of a probe, pushing the septum to one side, and the reflected light, the œdema and inflammatory state could be seen to subside at the junction of the cartilaginous and osseous portion of the septum. It looked as if the cartilage had been mashed down from above and spread out laterally, which, however, was not the case, since the fracture was vertical and occasioned evidently by the blow striking anteriorly. Naturally there was considerable tenderness. Some little bleeding had occurred from the left side. By palpating the protruding masses the evidence of some fluid could be distinguished, but by no means marked. Considerable bloody serum would exude when the septum was pushed to one side and a forcible expiration was made by the patient. The nasal bones were intact. The turbinates were in no wise enlarged or œdematous.

The treatment consisted in thoroughly cocainizing the parts in order to allow a more perfect examination, and at the same time to see if there would be any appreciable contraction of the tissues. Two firm plugs of absorbent cotton, saturated with a bichloride solution, were introduced anteriorly for the purpose of their exerting some pressure upon the sides of the septum. The patient was ordered a solution of aristol in liq. albolene, to be introduced anteriorly with a glass dropper and allowed to permeate the tissues.

May 19th. Condition about same. The presence of fluid was seemingly so slight that the plugs were again introduced in hopes of keeping the septum vertical and at the same time aiding absorption. Fortunately, with the fracture there was no decided septal deviation.

May 20th. Slight diminution in the size. The plugs were used during the day and removed in the afternoon.

May 21st. Left side looking much better where there was a freer escape of serum. Right side tender. Plugs used during the day.

May 22d. Swelling much less. Breathes slightly through both sides. Plugs soaked in vaseline were renewed. Vaseline was found to act much nicer, not producing that sobby appearance of a watery solution.

May 23d. Plugs discontinued. The passages are a little freer.

May 26th. About the same. Both sides sprayed with oil of eucalyptol in vaseline. The protuberances were painted with a solution of nitrate of silver (5 grs. to ℥).

May 27th. Very slight change. Plugs did not meet the desired result. Scarification was used on the right side.

May 28th. Mucous membrane on the left side of the septum thoroughly incised, followed by the evacuation of about two or three drachms of sero-pus. This was followed with a collapse of the membranes on both sides of the septum, and increased nasal respiration. Both sides thoroughly sprayed.

May 29th. Right side incised, followed with blood and serum. Aristol in liq. albolene (10 grs. to ℥) used throughout the treatment.

June 3d. Parts looking much better. Left side again incised. Septum painted on both sides with silver (10 grs. to ℥).

June 9th. Application of silver again made. Breathing much freer.

June 17th. Right side nearly normal. No septal deviation Union restored. Left side painted with silver (20 grs. to ℥).

June 23d. Both sides looking normal. Slight ridge on the left side following the line of fracture.

August 20th. Patient appeared at office on account of an interference with breathing on the left side.

Examination showed some thickening of the cartilage throughout its whole extent. The ridge running vertically at the line of fracture in the left fossa affords some resistance to the entrance of air. Secretion is prone to gather at that point. Inferior turbinate somewhat hypertrophied.

Had the case been left untreated, trusting to the *vis medicatrix naturæ*, the result would probably have been far different. That the case in time would have recovered and fairly good breathing obtained, at least on one side, there is no doubt, but the probabilities are that there would have been left a large enchondroma occluding the canal on that side, or a very severe septum deviation bringing the same results. Some very distinguished rhinologists believe that blows upon the nose are more often the etiological factors in producing septum deviations and spurs than any other cause, and I must confess that in all severe cases of this pathological condition which have come under my observation, I have been able to elicit from the patient the history of a blow during some period of his previous life. The term "pathological condition" has been used, a term which requires still further explanatory remarks, for I believe with many other observers that some deviation of the septum is the normal condition, and only reaches a pathological state when it interferes with the proper physiölogical functions of the nasal apparatus. Thus in the case herein described a few sprays and cautery of the turbinate were deemed sufficient without further radical measures. The case presented is an interesting one, as well as one of much uniqueness. I have been unable to find any similar or quasi-similar case mentioned in the literature at my disposal. Bosworth, in his late extensive work on the nose and throat, has nothing upon the subject. Traumatism of the nose is by no means an infrequent accident. From its decidedly protruding character and prominence in the make-up of the facial anatomy, one would suppose that it would most often be the objective point for the reception of blows directed and those unintentional. Such is probably the case, although no writer of whom I am cognizant has compiled statistics of the frequency of *nasalities* as compared with those of other portions of the face. A blow upon the nose is very distressing to the recipient, both *post hoc* and *propter hoc*, bringing tears alike to the happy as well as the morose. The physiology of the fifth cranial nerve, its relations and reflex actions, is an interesting study, but my object is no extended essay, but simply to add a few remarks to the case already presented. Any severe blow upon the nasal extremity is likely to cause some œdema and some interference with nasal breathing. This occurs whether the obstruction be confined to the outer wall of these cavities or to the septum itself. All the tissues of that portion known to the laity as the nose, i. e., the soft portion which gives character to the nasal expression, is apt to be involved. The mucous membrane covering the turbinate bones is of the character known as erectile tissue, that is, tissue which becomes enlarged by the engorgement of its capillary spaces. While this portion of the nose is not prominent enough for the direct reception of a blow, yet the transition of its mucous membrane with the skin is so intimate and the blood vessels of both so communicable that both are usually simultaneously involved. Any one who has ever given thought and study to the subject knows how prone to engorgement is the mucous membrane in this region, so that whenever the slightest opportunity offers itself for the fulfillment of this condition, it is seized with avidity. And hence arise our nasal stenosis in acute rhinitis. In the present case, however, the enlargement was confined entirely to the cartilaginous portion of the septum with no involvement of the external lateral walls of the cavity nor of the mucous membrane covering the bony portion of the septum. The blow in striking the septum anteriorly caused a bending and fracture vertically of the cartilage since its attachment to the bony portion posteriorly prevented the further transmission of the blow. The cartilage thus gave way at its weakest point, which in this case, as would naturally be supposed, was a point midway in the horizontal diameter. With this fracture came a rupture of the adjacent arterioles and mucous membrane, which thus caused the effusion of blood and subsequent bulging of the membrane. The protrusions from each side of the septum were of equal dimensions and communicable, as was demonstrated after the puncture. In the process of repair a ridge was left, running vertically, following the line of fracture, of a decided thickening throughout the whole cartilaginous portion of the septum, as the examination showed on August 20th. At that time the stenosis occasioned by the ridge was slight, so slight as to need no operative interference.

In the treatment of all such cases a palliative, rather than a radical, measure is the course to be pursued. Should the fracture be attended with decided inclination to one side or the other, pressure in some form should be made, in order that the final result may not be a deviated septum. The wearing of plugs of any description is exceedingly annoying, as any who has ever worn them can testify, and in case of fracture of the cartilaginous portion of the septum, they do not perfectly meet the end for which they were intended. That they do some good cannot be denied. One must remember that the nasal septum, anterior to the superior maxilla bones, has but a small triangular portion of cartilage to fill the space between the vomer and perpendicular plate of the ethmoid, as can readily be seen by examining a skull. All the front portion of the nose consists of soft tissues capable of distention, and the inefficacy of plugs to make pressure upon the septum was forcibly illustrated to me by the case in question, the whole blunt of the pressure being in the distention of the soft tissues laterally (externally). Should the mucous membrane of the septum be much swollen, the nature of the enlargement may be diagnosed by the introduction of the tips of the little fingers into each nasal cavity and the portion palpated. Should fluid be detected, the part should be thoroughly cocainized and a free incision be made down to the cartilage. This procedure should be done on only one side at a time, since the pain and soreness subsequent is a condition which sometimes follows. A warmed spray of eucalyptol in vaseline must be used throughout the course of treatment. On the next day the other side may be punctured if necessary, and if deviation exists the septum compressed with Bosworth's rhinoplastos. This latter plan is much more effective than plugs, and should be done every morning in order to allow the healing process to leave the septum in its normal position. Occasionally the deviation is so great that a septal clamp has to be worn for a couple of days. As soon as all serum or sero-pus, as in the case presented, has been permanently evacuated, the mucous membrane and hyperplastic state of the tissues should be contracted by the application of some astringent, preferably nitrate of silver (20 grs. to ℥). The resultant condition of the nasal cavaties after this plan of treatment will usually prove satisfactory. Later should secretions gather at any point and the mucous membrane look roughened, vaseline sprays applied night and morning will usually restore the parts to a normal condition.

14½ *Whitehall St.*

